# Rehabilitation of Posterior Maxilla with Zygomatic and Dental Implant after Tumor Resection: A Case Report

**DOI:** 10.1155/2013/930345

**Published:** 2013-02-28

**Authors:** Faysal Ugurlu, Coskun Yıldız, B. C. Sener, Atilla Sertgoz

**Affiliations:** Department of Oral and Maxillofacial Surgery, Dentistry Faculty, Marmara University, 34365 Istanbul, Turkey

## Abstract

Zygomatic implants have been used for dental rehabilitation in patients with insufficient bone in the posterior upper jaw, due to, for example, tumor resection, trauma, or atrophy. Zygomatic implants are an alternative to complex free or vascularized bone grafting and distraction osteogenesis. A 42-year-old male patient with a severe defect in the right posterior maxilla, starting from the first canine region, which had occurred after tumor resection 3 years earlier, was referred to our department. One zygomatic implant (Brenemark System, Nobel Biocare, Goteborg, Sweden) to the zygoma and one dental implant to the canine region were placed. After a 5-month osseointegration period, a fixed denture was fabricated and adapted to the implants. Although the surgical and prosthetic procedures for zygoma implants are not easy, the final outcomes can be successful with appropriate planning.

## 1. Introduction

Maxillary posterior defects that occur after tumor resection or trauma are challenging to reconstruct and rehabilitate. The aim of rehabilitation is not only to provide a cosmetically acceptable appearance, but also to restore oral functions, such as deglutition, mastication, and phonation. To provide better function, an implant-supported prosthesis can be fabricated. However, implant placement in the zygoma and pterygoid bone is difficult due to the variable anatomy and varying degrees of atrophy possible in the maxillofacial region [1, 2]. The surgery is not without risk because the drill path is close to critical anatomical structures, such as the maxillary sinus, the nasal cavity, and the eyes [2, 3]. A small angular error may result in significant positional errors at the end of the tool trajectory. Furthermore, the limited intraoperative visibility, especially given the anatomical intricacies of the curved zygomatic bone, makes this kind of surgery a demanding procedure.

In recent years, preoperative 3D planning of implant treatment that takes into account both the quality and quantity of the jawbone and prosthodontic considerations has had a major influence on final implant treatment outcome. In particular, before zygoma implant placement, 3D imaging and planning should be performed to ensure the safety of surgery and rehabilitation. This case study describes the clinical management of prosthetic rehabilitation of a patient who underwent zygoma implant placement.

## 2. Case Report

**Figure 1:**
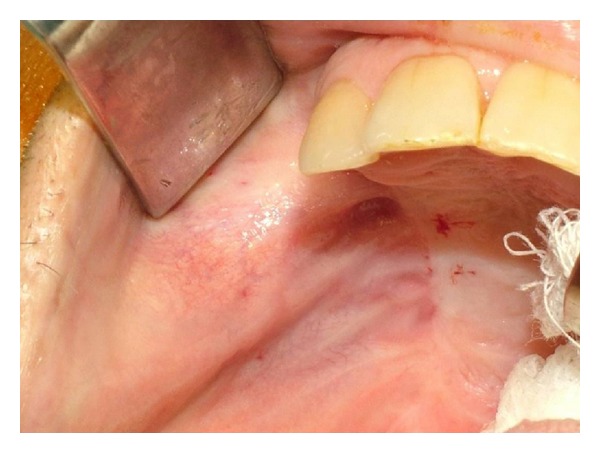
Preoperative intraoral view.

**Figure 2:**
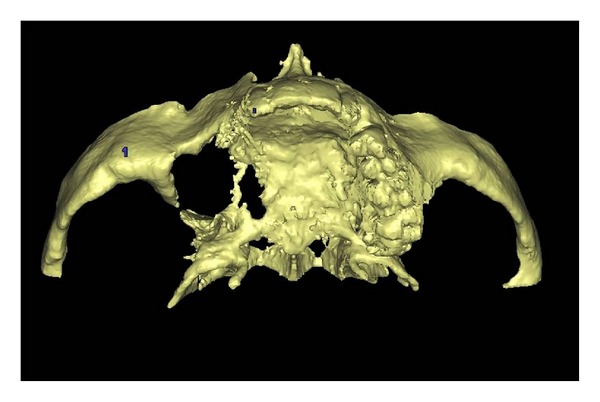
3D view of defect.

**Figure 3:**
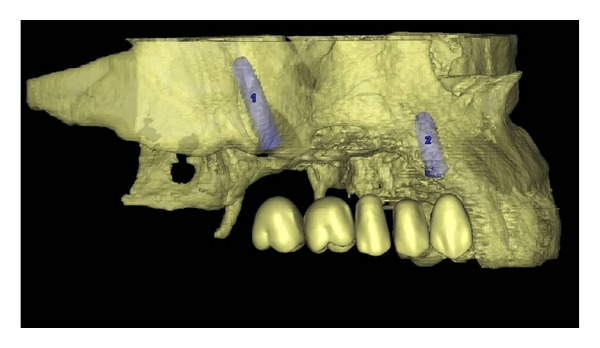
Preoperative simulation of implant planning.

A 42-year-old male patient was referred to our department with functional and esthetic issues. His anamnesis revealed that tumor resection had been performed to his right posterior maxilla 3 years earlier. His clinical examination revealed a severe defect in the right posterior maxilla, starting from the canine region (Figure 1). To be able to make an implant-supported fixed prosthesis, a zygoma implant was planned for the posterior maxilla, due to the huge bone defect in that area, and one dental implant was planned (Figures 2 and 3). The patient was evaluated preoperatively with respect to jaw size, bone volume, bone density, jaw relationships, intermaxillary distance, occlusal relation, and condition of the opposing dentition. Preoperative analysis of the anatomical conditions and possible maxillary pathology was evaluated using panoramic radiographs and a cone-beam CT scan.

**Figure 4:**
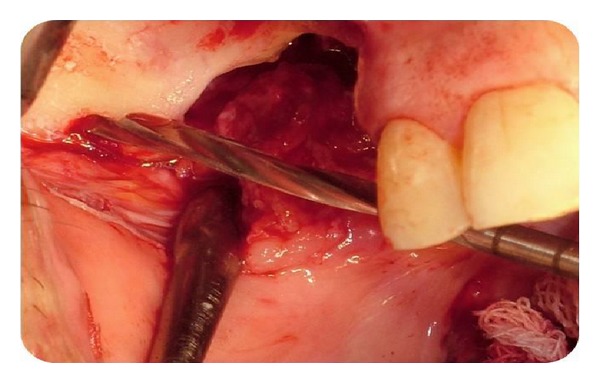
Intraoperative view of zygoma after flap elevation.

**Figure 5:**
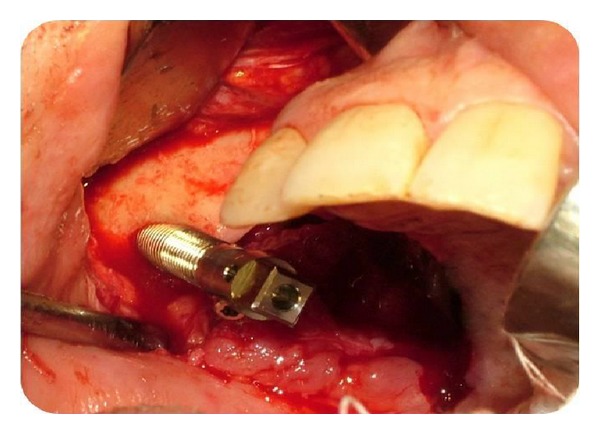
Implant placement of zygomatic implant.

The operation was performed under intravenous sedation. A crestal incision was made and a mucoperiosteal flap was elevated up to the zygomatic buttress (Figure 4). The zygoma implant was drilled and placed to the posterior; one dental implant was placed in the premolar region (Figure 5). The soft tissues were readapted and sutured back into position with silk sutures. An antibiotic (clindamycin, 600 mg/day) was prescribed for 10 days postoperatively. The remaining sutures were removed after 10 days.

**Figure 6:**
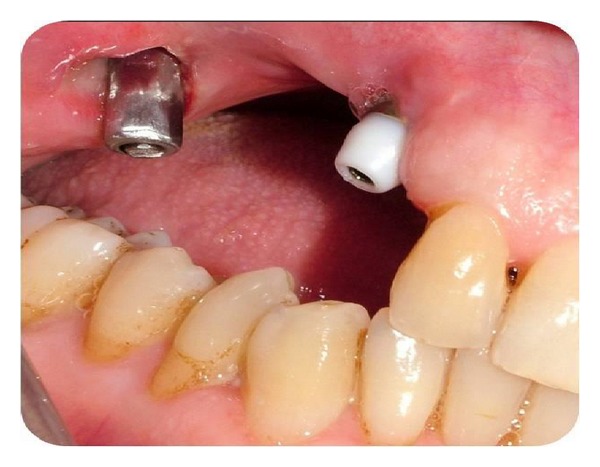
Intraoral view during osteointegration period.

**Figure 7:**
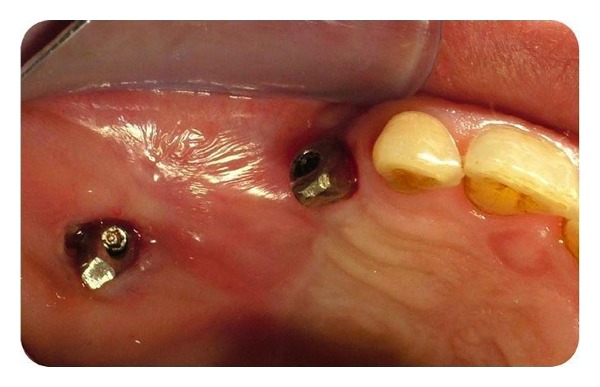
Intraoral view after implant abutments.

**Figure 8:**
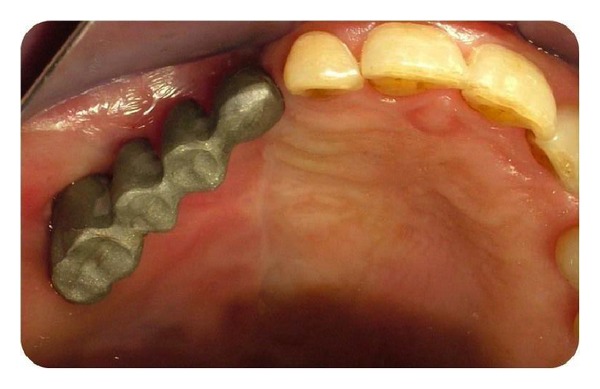
Fabricated Metal base of bridge.

Permanent prosthetic rehabilitation was initiated 5 months after implant placement (Figure 6). Metal impression copings were screwed onto the implants after removal of the healing screws. An impression was taken using an open-tray technique. Metal-supported porcelain restorations were constructed using conventional methods (Figures 7 and 8).

**Figure 9:**
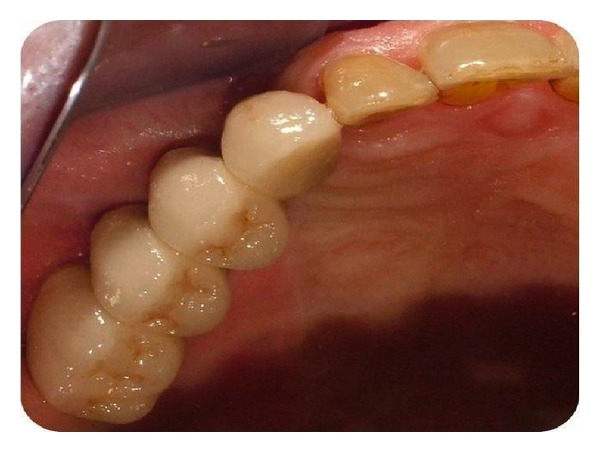
Metal supported porcelain bridge.

**Figure 10:**
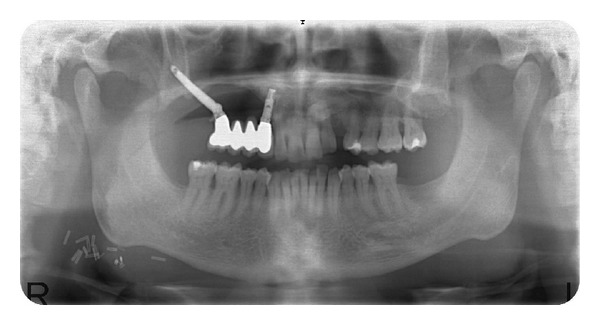
Radiographic view after 1 year follow-up.

The patient was followed up at 5 years after the prosthetic rehabilitation. The last clinical and radiographic assessments were uneventful, and the patient's satisfaction with the esthetic result was excellent (Figures 9 and 10).

## 3. Discussion

Reconstruction of posterior maxillary defects is a challenge. Many procedures, such as onlay grafts [4], free or microvascular bone grafts [5, 6], transport distraction osteogenesis [7], and apposition grafts with or without a Le Fort I osteotomy [8] are well documented and have success rates of 60–90%. However, these often involve invasive and lengthy surgeries, long treatment time, and some morbidity. Microvascular bone grafts are complex and risky operations. In addition, these conventional techniques cause donor site morbidity during harvesting of soft tissues and bone grafts [4]. Furthermore, free bone grafts are commonly associated with unpredictable resorption during healing [4, 5, 9].

Zygomatic implants, an alternative to these techniques, have survival rates of 98–100% [10]. Although no randomized clinical trial that compared sinus grafts or onlay grafts and zygomatic implants has been reported, the survival rates suggest that zygomatic implants should be considered an alternative to bone grafting and applied in routine rehabilitation of the totally edentulous patient with high resorption of the maxilla and severe defects in the posterior maxilla after tumor resection. However, the placement of zygoma implants is not without risk, due to the anatomically complex operation site. Implant-guided surgery is a suitable method for minimizing the risks and improving the precision of the surgery. This method enables the transfer of the preoperative plan accurately to the operating theatre.

The drill guides dictate the location, angle, and depth of insertion of the implant, so as to provide a link between the planning and the actual surgery by transferring the simulated plan accurately to the patient [11, 12]. Another possibility to establish the relationship between the operation site and the computer-generated additional information is the use of tracking technology, which continually registers the position of the patient and surgical tools by means of special sensors (“computer-aided navigation”) [13]. This navigation technology has now been tested and demonstrated by many research groups and clinicians for use in many applications [11–13]. However, these methods are expensive to use.

With a thick cortical layer, the zygoma bone offers a solid and extended anchorage that can support the masticatory forces applied at the occlusal level. The amount of bone volume in the zygoma has been reported in several studies. These concluded that the high volume of bone and the possibility of tricortical anchorage increased both the success and survival rate of zygomatic implants [14, 15].

The greatest advantage of zygomatic implants is elimination of donor site morbidity and infection in the graft material. Also, treatment time is decreased significantly by avoiding the need for bone healing after grafting. Also, their use reduces the number of surgical operations required and so also the associated hospital costs [16].

Despite their advantages, zygomatic implants also have many complications and problems after implantation. Soft tissue inflammation around the abutments has been reported in some studies of zygomatic implants [17]. The depth of the palatal mucosa at the level of the posterior implant is normally 5 mm, consisting of parakeratinized epithelium that is not comparable with the normal pocket [18]. Lack of soft tissue with the bone defect can lead to gingival problems around the implant during or after osseointegration, as in our case. To overcome this, oral hygiene levels must be high. With good oral hygiene and close follow-up (every 3 months) no inflammation was recorded around the zygoma implant in the present case.

Sinusitis has been reported by a number of authors [19, 20]. The incidence was 14–30%. In some cases, patients with an oroantral fistula may develop suppuration with or without sinusitis. Treatment involves the administration of antibiotics and/or repositioning soft tissue, without removal of the stable zygomatic implant. Causes of sinusitis include perforation of the sinus membrane and leakage at the level of the maxilla due to a hole in the zygomatic implant, leading to migration of bacteria from the mouth to the sinus [16]. In the present case, no sinusitis or oroantral fistula occurred.

The zygomatic implant-supported prosthesis requires special care due to the biomechanical forces that affect the long-term stability of an implant-supported restoration. Use of a stiff prosthesis is necessary because flexing of the materials can cause deformation and deviation, resulting in loss of implant or loosening of the junction between the prosthesis and fixation. The success rates of implants in the zygomatic bone vary from 95% to 97% with 12–124 months of follow-up observation [21, 22], and the patient satisfaction rate is 80% 1 year after installation of the prosthesis [23].

## 4. Conclusions

Zygoma implants are a unique alternative for rehabilitation of the posterior maxilla after tumor resection or trauma. Due to both the anatomical intricacies of the zygomatic bone and the implant length, the placement of zygoma implants still represents a challenge to prosthodontists. To minimize the risks of surgery, 3D reconstruction, preoperative planning, registration, surgical implant guidance, and a motion tracking algorithm should be used.
